# A Japanese version of the Perceived Stress Scale: cross-cultural translation and equivalence assessment

**DOI:** 10.1186/1471-244X-8-85

**Published:** 2008-09-30

**Authors:** Chizu Mimura, Peter Griffiths

**Affiliations:** 1 Visiting Research Associate, Florence Nightingale School of Nursing and Midwifery, King's College London, UK; 2 5th Floor Housei Dai-ichi Building, 1-20-10 Sigamo, Toshima-ku, Tokyo, Japan; 3 National Nursing Research Unit, King's College London, James Clerk Maxwell building, Waterloo Road, London, UK

## Abstract

**Background:**

This paper describes the development of a Japanese version of the Perceived Stress Scale (PSS), and examines the equivalence between the original and translated version. The PSS is one of the few instruments to measure a global level of perceived stress, and has been widely used in a range of clinical and research settings. The PSS has already been translated into several languages, but there is no validated Japanese version.

**Methods:**

A forward-backward procedure was implemented. Multiple forward and backward translations were produced, and a panel of reviewers verified conceptual and semantic equivalence between the source and final versions. Non-professional translators who were not brought up in bilingual families were used in order to enhance representativeness of language in the target populations. The PSS was administered to 222 native English speakers and the Japanese version (PSS-J) to 1320 native Japanese speakers.

**Results:**

Factor analysis showed similar factor loadings of the items and satisfactory factorial agreement between the PSS and PSS-J. Cronbach's alpha coefficient was high for both versions and for each factor.

**Conclusion:**

It is concluded that the PSS and PSS-J are substantially equivalent and suited for use in comparative cross-cultural studies.

## Background

This paper reports the translation of the Perceived Stress Scale (PSS) [1] from English to Japanese and equivalence assessment between the original PSS and the translated version (PSS-J) for use in a cross-cultural study of occupational stress amongst trainee health professionals.

There are many approaches to measuring stress and in any given study it is important to consider conceptual, practical and scientific aspects [2]. Many theorists have provided three broad categorisations of stress (e.g., [3-6]). A strong commonality among these categorisations is that the first concept puts the emphasis on stressful events, the second on consequences of stress and the third on individual appraisals of situations. However, there is a growing consensus that stress arises from an imbalance between the individual's perception of demand charged by the situation and his/her estimate of the ability to cope with the demand [4]. That is to say the experience of stress is dependent upon the individual appraisal of external stressor and his/her own capability. In the context of a cross-cultural study, conceptualising stress by focusing on specific stressful events and/or particular responses runs the risk of being inherently culture bound in measuring stress because it requires a common perception of that which is potentially stressful, a common opportunity of experiencing particular situations and a common attitude of responding to external stressors.

The PSS (see Additional file 1) is one of the few instruments to measure a global level of perceived stress, dealing with the degree to which situations in one's life are appraised as stressful as opposed to the presence of particular stressors. It is a 14-item instrument that assesses perceived stressful experience or stress responses over the previous month using 5-point Likert type scales. Total possible scores are from 0 to 56. Higher scores represent high stress levels. The PSS is a well established measure. The creators reported convergent validity indicated by relationships with depressive (r = .76, n = 332) and physical (r = .70, n = 64) symptomatology scales. Internal consistency reliability was high with Cronbach's alpha coefficient ranging from .84 (n = 332) to .86 (n = 64) [1]. It has been used in a range of settings and shown to relate to a number of physiological and psychological correlates of stress [7-11]. The PSS has been utilised for evaluating the effect of interventions to reduce stress [12] and has been used as a reference standard for examining validity of new stress measures [13].

The PSS has already been translated into several languages including Spanish [14], Swedish [15] and Chinese [16]. There is no validated Japanese version of the PSS although subsequent to the commencement of this study Japanese translations of the scale have been reported but not subjected to validation ([17] for example). Thus it has particular value in cross-cultural studies to develop a Japanese version of the PSS since it has been used in a wider range of cultures than most measures. Therefore, we developed the PSS-J (see Additional file 2). A small pilot study [18] suggested the reliability and equivalence in terms of factor structure between the PSS and the PSS-J although the sample size was too small to draw firm conclusions. The present study further tested these properties using a much larger sample. Permission to translate the PSS into Japanese was granted by the developer, Professor Sheldon Cohen. The study was conducted under the aegis of a wider study, which had been ethically scrutinised and approved by the authors' institutional ethical committee.

## Methods

### Translation

The translation procedures were informed by the European Research Group on Health Outcome recommendations [19] and the International Test Commission Guidelines [20]. The repeated forward-backward translation procedure was adopted as the most suitable strategy that was pragmatically possible.

In Phase 1, four married couples of British and Japanese origin were separately asked to translate the original scale into Japanese with each couple among themselves discussing the conceptual, semantic and content equivalence between the original and their translation. The four couples were selected in accordance with the following criteria:

(1) one member of the couple was a native English speaker and the other a native Japanese speaker;

(2) both members were reared and educated either in English in an English-speaking country or in Japanese in Japan until at least 18 years of age;

(3) they have spent more than five years together since they married.

These criteria were used to identify translators who were familiar with both their own language and cultural background and that of the alternative language. The use of married couples was based on the opportunity such couples presented for exchanging a native speaker's insight into expressions in different languages among an intimate couple without the bias of representativeness introduced by restricting translators to those with a formal academic training. None of the individuals involved were professional translators. Thus it was hoped that an equivalent translation would be produced that was potentially more representative of the wider cultures than would be gained from a bilingual person or highly trained translators. All four couples happened to be of a British male and a Japanese female. They were fully informed of the objectives of their role in the whole procedure and were asked to discuss conceptual, semantic and content equivalence and to emphasise meaning rather than word-to-word translation. One of the authors (CM whose first language is Japanese) unified the four Japanese translations created by this process into a single translated version. Selection among alternative Japanese translations was based upon the perceived "naturalness" of linguistic expression in the Japanese language version.

In Phase 2, an additional couple was identified using the same criteria. They were asked to back-translate the Japanese version produced in Phase 1 without sight of the original version. In Phase 3, five university lecturers at the authors' college (native English speakers) compared the original scale and the back-translation brought about by Phase 2, and checked for semantic discrepancies. In Phase 4, the author altered the Japanese expression of the parts found to be problematic in Phase 3 with reference to any alternatives rejected in Phase 1. An example of differing translation when put back into English was Item 7 "how often did you feel that things were going as you expected in this past month?" as opposed to the original statement "in the last month, how often have you felt that things were going your way?" All of the lecturers pointed out that the translation could be negative but the original was more positive, thus the Japanese translation was altered so that it did not include negative meanings.

The couple used in Phase 2 re-translated them into English. One of the panel used in Phase 3 checked discrepancies between the original scale and the re-translation. Detailed discussion of cultural difference and nuance aimed to ensure semantic equivalence and to overcome conceptual differences by identifying parallel concepts. This process was repeated until problems were resolved.

### Equivalence assessment

#### Respondents

Data were collected in the UK using the original English language PSS and in Japan using the translated version which we refer to as the PSS-J. Participants were recruited from full-time BSc nursing and pharmacy students of all years (1 to 4) at single university institutions in central London and Tokyo. Non-native English/Japanese speakers were excluded as appropriate to the version of the scale being tested. Data were obtained from 131 nursing and 91 pharmacy students in the UK (n = 222) of whom 28 were male (12.6%) and 194 were female (87.4%). Ages ranged from 18 to 45 and the mean age was 22.1 (SD = 4.5). The Japanese sample comprised 344 nursing and 976 pharmacy students (n = 1320) of whom 296 were male (22.4%), 1018 were female (77.1%) and 6 (0.5%) did not indicate their gender. Ages ranged from 18 to 44 and the mean was 20.6 (SD = 2.8). The differences in sample size were largely dictated by the size of the student cohorts in each institution. The response rate was 70.3% in the UK and 83.6% in Japan.

#### Data Collection

The questionnaire was administered to the students in a class setting. After permission for access to the students was obtained from the head of department and the course leader, the investigator visited the class in a room before or after a lecture. The questionnaires were distributed only to students who agreed to participate in the study. For the pharmacy students in the UK, it was not possible for all students to complete the questionnaires immediately owing to their tight academic time schedule. Therefore, a designated box was allocated in their school, and they could choose to complete the questionnaire immediately or to return it in the box later. For all other groups questionnaires were gathered in the envelopes provided immediately after they finished completing the questionnaire in the room.

Prior to the data collection, a pilot study was conducted to check the feasibility of the administration process and the credibility of the original and translated instruments. The questionnaire was administered on nursing students undertaking their postgraduate courses in the same university as the main study (n = 38 in the UK, n = 23 in Japan). The procedure of data collection in the pilot studies was exactly the same as in the main study. No problem arose regarding the administration process, including in the data collection and data handling procedure in both countries. Respondents in the UK appeared sometimes to miss negative words such as "no," "not" and "unable" when reading the items and scored them in reverse. Thus, amendments were made to the questionnaire, emboldening and underlining negative words. As for the Japanese version of the questionnaire, no problem was observed, and therefore no amendment was made to it.

### Data Analysis

Factor structure was assessed by using exploratory factor analysis. With principal component analysis, the largest two factors were extracted and subjected to Varimax rotation with Kaiser normalisation. For the purpose of establishing equivalence, a two-factor solution was used as this had been identified in the original PSS [21-24]. After the analysis was carried out, target rotation [25] was performed to estimate factorial agreement of the two factors of the PSS and PSS-J for the different culture groups, which determine the construct equivalence. Cronbach's alpha coefficient was calculated to examine internal consistency reliability for the PSS and PSS-J and for each factor of the two scales.

## Results

### Descriptive findings

The mean score for the PSS was 27.6 (*SD *= 8.42), median was 27.0, and the range was from 7 to 51. The item mean score varied between 1.5 (*SD *= .90: Item 6) and 3.1 (*SD *= .83: Item 12). For the PSS-J, the mean score for the PSS was 29.3 (*SD *= 6.46) and median was 29.0, ranging from 6 to 53. The item mean score varied from 1.3 (*SD *= 1.06: Item 2) to 2.7 (*SD *= .87: Item 6)

### Factor structure

All the diagnostic tests indicated adequacy of proceeding with factor analysis. Bartlett's test of sphericity showed a significant result (p < .001) for both the PSS and PSS-J. For the Kaiser-Meyer-Olkin test (KMO) and the individual measure of sampling adequacy (MSA), a value of greater than 0.6 represents an acceptable result [26]. KMO was .92 and MSA ranged from .68 to .94 for the PSS. With respect to the PSS-J, these were .83 and from .78 to .89 respectively.

An inspection of the distribution of the eigenvalues confirmed a two-factor solution. The differences in eigenvalues between the second and third factors are relatively large compared to the rest (Table 1), which suggested that there were actually only two significant factors.

**Table 1:** Initial Eigenvalues explained by factors

	**PSS^a)^** **(n = 222)**			**PSS-J^b)^** **(n = 1320)**		
***Component***	*Total*	*% of Variance*	*Cumulative %*	*Total*	*% of Variance*	*Cumulative %*

1	5.98	42.73	42.73	3.34	23.84	23.84
2	1.54	10.99	53.72	2.63	18.75	42.59
3	0.95	6.77	60.49	1.04	7.40	49.99
4	0.91	6.52	67.01	0.97	6.90	56.89
5	0.70	4.99	72.00	0.80	5.71	62.60
6	0.60	4.29	76.29	0.76	5.40	68.00
7	0.54	3.86	80.15	0.71	5.05	73.05
8	0.51	3.61	83.76	0.66	4.72	77.77
9	0.44	3.15	86.91	0.61	4.33	82.10
10	0.43	3.06	89.97	0.55	3.94	86.04
11	0.40	2.88	92.85	0.53	3.76	89.80
12	0.37	2.61	95.46	0.50	3.59	93.39
13	0.33	2.37	97.83	0.49	3.52	96.91
14	0.30	2.17	100.00	0.43	3.09	100.00

Note: Extraction method – principal component analysis

a): The original Perceived Stress Scale, administered to native English speakers

b): Translated Japanese version of the Perceived Stress Scale, administered to native Japanese speakers

The results of the two-factor solution are presented in Table 2. In terms of the original PSS, the extracted two factors explained 53.7% of the variance in which the first factor accounted for 42.7% and the second factor for 11.0%. The items stating negative attitude largely loaded highly on the first factor except for Item 13. Items 7 and 10 also loaded highly on the first factor in spite of items stating positive experience, but these two items had substantial correlation with both the factors.

**Table 2:** Factor analysis and reliability coefficient

			**PSS^a)^** **(n = 222)**		**PSS-J^b)^** **(n = 1320)**	
			***Factor***		***Factor***	

***Item***	***Statement***	***Attitude***	***I***	***II***	***I***	***II***

1	In the last month, how often have you been upset because of something that happened unexpectedly?	-	0.58	0.17	-0.04	0.63
2	In the last month, how often have you felt that you were unable to control the important things in your life?	-	0.77	0.27	0.11	0.67
3	In the last month, how often have you felt nervous and/or stressed?	-	0.78	0.08	0.03	0.73
4	In the last month, how often have you dealt successfully with irritating life hassles?	+	-0.03	0.80	0.64	-0.25
5	In the last month, how often have you felt that you were effectively coping with important changes that were occurring in your life?	+	0.21	0.73	0.72	-0.04
6	In the last month, how often have you felt confident about your ability to handle your personal problems?	+	0.47	0.65	0.73	0.01
7	In the last month, how often have you felt that things were going your way?	+	0.56	0.43	0.65	0.16
8	In the last month, how often have you found that you could not cope with all the things that you had to do?	-	0.74	0.23	0.01	0.40
9	In the last month, how often have you been able to control irritations in your life?	+	0.41	0.63	0.60	0.04
10	In the last month, how often have you felt that you were on top of things?	+	0.67	0.42	0.75	0.15
11	In the last month, how often have you been angered because of things that happened that were outside of your control?	-	0.71	0.19	0.08	0.61
12	In the last month, how often have you found yourself thinking about things that you have to accomplish?	-	0.44	-0.31	-0.06	0.60
13	In the last month, how often have you been able to control the way you spend your time?	+	0.52	0.14	0.40	0.29
14	In the last month, how often have you felt difficulties were piling up so high that you could not overcome them?	-	0.78	0.17	0.14	0.71
	% of Variance		42.7	11.0	23.8	18.8
	Cronbach's alpha coefficient		0.88	0.77	0.76	0.75
			0.89		0.74	

Note: Extraction method – principal component analysis

Rotation method – Varimax with Kaiser normalisation

a): The original Perceived Stress Scale, administered to native English speakers

b): Translated Japanese version of the Perceived Stress Scale, administered to native Japanese speakers

For the translated PSS-J, the rotated two factors accounted for 42.6% of the variance. The first largest factor explained 23.8% and the second factor 18.8% of the variance. All items stating a positive attitude had high loading on the first factor and items of negative experience had high loading on the second factor.

In the target rotation, the factor solution of the PSS was rotated to the loadings of the PSS-J. The identity coefficient was .90 for the first largest factor and .93 for the second largest factor. The proportionality coefficient was .90 and .94 respectively. These coefficients indicate factor congruence if figures are .9 or higher [25]. Therefore, it can be deducted that the two factors of the PSS and PSS-J were equivalent for the two groups.

### Internal consistency reliability

For the PSS, Cronbach's alpha was .89 for the whole scale, .88 for the first largest factor and .77 for the second largest factor. For the PSS-J, it was .74, .76 and .75 respectively. Cronbach's alpha in excess of .7 is generally considered to be acceptable for a scale [2]. Thus, these results were satisfactorily high (see Table 2).

The item-total correlation for the PSS was largely strong, with relatively weak correlation for Item 4 (*r *= .39, p < .01) and Item 12 (*r *= .27, p < .01), and the other items ranging from *r *= .53 (p < .01) to *r *= .74 (p < .01). The inter-item correlation ranged from *r *= .03 (statistically not significant) to *r *= .61 (p < .01), with most inter-item correlations indicating *r *= .40 or higher. For the PSS-J, The item-total correlation was from *r *= .42 (p < .01) to *r *= .62 (p < .01) except for Item 4 (*r *= .27, p < .01) and Item 8 (*r *= .36, p < .01). The inter-item correlation was mostly weak raging from *r *= .002 (statistically not significant) to *r *= .48 (p < .01)

## Discussion

The factor analysis revealed that for the original PSS, all the items of negative experience loaded highly on the first factor. Although some items reflecting positive experience were also strongly related to the first factor, these items also had substantial correlation with the second factor. This pattern of factor structure is nearly identical to that identified by previous empirical research. Cohen and Williamson [21] showed a two-factor structure for the PSS in a US sample (n = 2387) and each factor reflected positively or negatively phrased items. Subsequent studies confirmed this factor structure in psychiatric patients in Canada (n = 96) [24] and in psychology students in Mexico (n = 365) [22]. In the current study as well, the first factors can be labelled as "negative perception" and the second factor as "positive perception." As mentioned above, however, Items 7, 10 and 13 highly loaded on the first factor in spite of positive statements. This might be due to a relatively small sample size (n = 222) for factor analysing the 14-item scale. In the study by Hewitt et al. (n = 96) [24], factor loading was not reported about Items 10, 12 and 13 which might be differently grouped in terms of factorial nature.

On the other hand, as for the PSS-J, all items stating positive attitude were highly related to the first factor and all items of negative attitude to the second factor. Thus, labels for these factors can be "positive perception" and "negative perception" respectively.

The variance explained by the factor is somewhat different between the two cohorts in the current study, and also from that found in these previous studies. The first largest factor explained 42.7% and the second factor 11.0% in the UK sample, and 23.8% and 18.8% respectively in the Japanese sample in the current study. Previously reported variance was: 25.9% accounted for by the first factor and 15.7% by the second factor in the US sample [21]; 31.4% and 15.2% respectively in the Canadian sample [24]; and 32.6% and 15.4% in the Mexican sample [22]. Such dispersion might be derived from cultural differences and different sample size.

Comparing the factor loading of the PSS and the PSS-J, although the magnitude of each factor was different, items stating positive attitude were gathered in the "positive perception" factor and items of negative attitude were in the "negative perception" factor for both scales. Also, the factor congruence coefficient indicated satisfactory factor agreement between the PSS and the PSS-J. It can be seen that the PSS and PSS-J were similar in factor structure. Cronbach's alpha for each factor was high. This suggested that all factors were internally consistent. The equivalence between the PSS and PSS-J was supported through a similar factor structure and factor loading on items. However, as pointed out by Cohen and Williamson [21] and González and Landero [22], the distinction between the two factors is considered irrelevant and total scores obtained by summing responses to all 14 items should be used for the purpose of measuring perceived stress.

Regarding both the PSS and PSS-J, all the items except for one (Item 12 in the PSS; Item 4 in the PSS-J) indicated acceptable item-total correlations. Generally, items showing an item-total correlation of 0.3 or lower are considered to be dropped from the scale [27]. However, a scale with an acceptable Cronbach's alpha may still have one or more items with low item-total correlations [27]. Thus, the findings of the current study were indicative of sufficient item-total reliability for the PSS and PSS-J.

As described in the translation section, a number of efforts were made to produce a Japanese version of the PSS as equivalent as possible to the original scale. Using intimate couples of a native English speaker and a native Japanese speaker probably contributed greatly to addressing problems that are likely to occur when an instrument is translated into other languages. These would include conceptual problems such as differences in conceptualisation and behaviours associated with the construct of a scale and inappropriateness of item content, and linguistic problems such as erroneous literal translation and poor wording [19,20]. Multiple forward and backward translations and verification of the equivalence between the source and final version by a multi-disciplinary reviewer panel would also resolve these issues. However such couples are likely to differ from the population in general. The translation might, therefore, be biased although professional translators and those who generate the original items on such scales are equally unlikely to represent the general population.

While the sample size was large, another limitation of this assessment is that the subjects consisted of undergraduate nursing and pharmacy students who were recruited from a single institution in each country. Also, they were predominantly female. The findings may, therefore, be influenced by stress characteristics unique to them such as gender, stressors as a consequence of actually being undergraduates or particular factors relating to the subject of study. Further equivalence assessment using a sample that is more representative of the population in general would ideally be conducted to overcome these limitations. Certainly it is important that research using this scale in new population assesses the factor structure and internal consistency for its own sample.

## Conclusion

In addition to the small-scale preliminary test [18], this study further supported the equivalence between the PSS and PSS-J through analysing factor loadings on items, factorial agreement and internal consistency. We conclude that the PSS-J is a suitable tool for the study of perceived stress among native Japanese speakers and that there is sufficient evidence of the equivalence of the PSS and PSS-J to consider them as equivalent in cross-cultural studies.

## Competing interests

The authors declare that they have no competing interests.

## Authors' contributions

CM conceptualised and designed the study, coordinated the translation process, collected and analysed the data, interpreted the results and drafted the manuscript. PG participated in its design and translation, supervised the analysis and interpretation, and helped to draft and revise the manuscript. All authors read and approved the final manuscript.

## Pre-publication history

The pre-publication history for this paper can be accessed here:



## Supplementary Material

Additional file 1 The original version of the Perceived Stress Scale (PSS) Click here for file

Additional file 2 The Japanese version of the Perceived Stress Scale (PSS-J) Click here for file
