# Supplementary material for: A Japanese version of the Perceived Stress Scale: cross-cultural translation and equivalence assessment
Source: BMC Psychiatry. 2008 Sep 30;8:85. doi: 10.1186/1471-244X-8-85 (PMC2569029; doi:10.1186/1471-244X-8-85)
Supplement: Additional file 1 — The original version of the Perceived Stress Scale (PSS) [file 1471-244X-8-85-S1.pdf]

Original Perceived Stress Scale

Below are listed a number of ways that one person could feel or behave.
For each statement, indicate how often you have felt or behaved in such a way in the last month.
If you never felt or behaved circle N, if almost never circle AN, if sometimes circle S,
if fairly often circle FO and if very often circle VO.

	Never	Almost never	Some-times	Fairly often	Very often
In the last month, how often have you been upset because of something that happened unexpectedly?	N	AN	S	FO	VO
In the last month, how often have you felt that you were **<u>unable</u>** to control the important things in your life?	N	AN	S	FO	VO
In the last month, how often have you felt nervous and/or stressed?	N	AN	S	FO	VO
In the last month, how often have you dealt successfully with irritating life hassles?	N	AN	S	FO	VO
In the last month, how often have you felt that you were effectively coping with important changes that were occurring in your life?	N	AN	S	FO	VO
In the last month, how often have you felt confident about your ability to handle your personal problems?	N	AN	S	FO	VO
In the last month, how often have you felt that things were going your way?	N	AN	S	FO	VO
In the last month, how often have you found that you could **<u>not</u>** cope with all the things that you had to do?	N	AN	S	FO	VO
In the last month, how often have you been able to control irritations in your life?	N	AN	S	FO	VO
In the last month, how often have you felt that you were on top of things?	N	AN	S	FO	VO
In the last month, how often have you been angered because of things that happened that were outside of your control?	N	AN	S	FO	VO
In the last month, how often have you found yourself thinking about things that you have to accomplish?	N	AN	S	FO	VO
In the last month, how often have you been able to control the way you spend your time?	N	AN	S	FO	VO
In the last month, how often have you felt difficulties were piling up so high that you could **<u>not</u>** overcome them?	N	AN	S	FO	VO
