# Supplementary material for: A Japanese version of the Perceived Stress Scale: cross-cultural translation and equivalence assessment
Source: BMC Psychiatry. 2008 Sep 30;8:85. doi: 10.1186/1471-244X-8-85 (PMC2569029; doi:10.1186/1471-244X-8-85)
Supplement: Additional file 2 — The Japanese version of the Perceived Stress Scale (PSS-J) [file 1471-244X-8-85-S2.pdf]

日本語版 Perceived Stress Scale

下の表は，人がどのように感じたり振るまったりするかという例を挙げたものです。
それぞれの項目について，そのようなことが，この１ヶ月間にどのくらいあったかを印して下さい。
全くなかった場合は「無」に，ほとんどなかった場合は「稀」に，ときどきあった場合は「時々」に，
よくあった場合は「よく」に，何度もあった場合は「頻」に○をつけて下さい。

	全くなし	ほとんどなし	ときどき	よくあった	何度もあった
この１ヶ月間，思いがけない事が起きて気が動転したことがどのくらいありましたか。	無	稀	時々	よく	頻
この１ヶ月間,あなたの人生にとって重要な事柄を自分でコントロールすることが出来なかったと感じたことがどのくらいありましたか。	無	稀	時々	よく	頻
この１ヶ月間，神経質になっている，あるいはストレスがたまっていると感じたことがどのくらいありましたか。	無	稀	時々	よく	頻
この１ヶ月間，イライラするような問題をうまく処理したことがどのくらいありましたか。	無	稀	時々	よく	頻
この１ヶ月間，生活の中で起きた大きな変化に上手に適応していると感じたことがどのくらいありましたか。	無	稀	時々	よく	頻
この１ヶ月間，自分の個人的な問題を扱う能力に自信を感じたことがどのくらいありましたか。	無	稀	時々	よく	頻
この１ヶ月間,自分にとって良い方向に事が運んでいると感じたことがどのくらいありましたか。	無	稀	時々	よく	頻
この１ヶ月間，しなければならないことに全部はうまく取り組めていないということがどのくらいありましたか。	無	稀	時々	よく	頻
この１ヶ月間，生活の中のいら立たしいことをコントロールすることがどのくらい出来ましたか。	無	稀	時々	よく	頻
この１ヶ月間，物事をうまく統制していると感じたことがどのくらいありましたか。	無	稀	時々	よく	頻
この１ヶ月間，あなたのコントロールのきかない範囲で起きた事に対して怒りを感じたことがどのくらいありましたか。	無	稀	時々	よく	頻
この１ヶ月間，ふと気がつくと，成しとげなければならない事柄に考えをめぐらせていた，ということがどのくらいありましたか。	無	稀	時々	よく	頻
この１ヶ月間，自分の時間の過ごし方をどのくらい自分でコントロール出来ましたか。	無	稀	時々	よく	頻
この１ヶ月間，大変な事があまりにも積み重なってしまって，乗り越えることが出来ないと感じたことが，どのくらいありましたか。	無	稀	時々	よく	頻
